# Influence of Aging on Corrosion Behaviour of the 6061 Cast Aluminium Alloy

**DOI:** 10.3390/ma14081821

**Published:** 2021-04-07

**Authors:** Ting He, Wei Shi, Song Xiang, Chaowen Huang, Ronald G. Ballinger

**Affiliations:** 1 College of Materials and Metallurgy, Guizhou University, Guiyang 550025, China; hthjyhchg@163.com (T.H.); sxiang@gzu.edu.cn (S.X.); cwhuang@gzu.edu.cn (C.H.); 2 Key Laboratory for Mechanical Behavior and Microstructure of Materials of Guizhou Province, Guizhou University, Guiyang 550025, China; 3 H.H. Uhlig Corrosion Laboratory, Massachusetts Institute of Technology, Cambridge, MA 02139, USA; hvymet@mit.edu

**Keywords:** As-cast 6061 aluminium alloy, localised corrosion, AlFeSi, Mg_2_Si

## Abstract

The influence of AlFeSi and Mg_2_Si phases on corrosion behaviour of the cast 6061 aluminium alloy was investigated. Scanning Kelvin probe force microscopy (SKPFM), electron probe microanalysis (EPMA), and in situ observations by confocal laser scanning microscopy (CLSM) were used. It was found that Mg_2_Si phases were anodic relative to the matrix and dissolved preferentially without significantly affecting corrosion propagation. The AlFeSi phases’ influence on 6061 aluminium alloy local corrosion was greater than that of the Mg_2_Si phases. The corroded region width reached five times that of the AlFeSi phase, and the accelerating effect was terminated as the AlFeSi dissolved.

## 1. Introduction

Al–Mg–Si alloys with excellent corrosion resistance, weldability, and formability, are widely used in transportation and other fields [1,2]. In Al–Mg–Si alloys, Mg_2_Si phases improve the hardness and mechanical performance [3,4], while Cu- and Fe- rich intermetallic phases were the inevitable inclusion origin from the smelting process [5,6,7]. Thus, there has long been a debate on which phase dominates the localised corrosion of Al–Mg–Si alloys. Some researchers have suggested that anode phase (Mg_2_Si) accelerates the corrosion rate as it dissolves in the substrate, El-Meshawy et al. [7] found that the AA6061 corrosion type and electrochemical corrosion parameters depended on its ageing conditions. On the other hand, some researchers concluded that the cathode phase (Fe-rich inclusions) prompts the substrate anode current. Using galvanic theory, Zander et al. [5] found that pitting penetration followed the deformation-induced AlFeSi (Mn, Cu) phase distributions. Both accelerating mechanisms were reasonable from an electrochemistry point of view, however, it was unclear which kind of phase substantially dominated the Al–Mg–Si alloys localised corrosion, and what contribution each phase made in Al–Mg–Si alloys localised corrosion.

Al–Mg–Si alloys are the most used high-strength aluminium alloys and contain primarily nanosized Mg_2_Si dispersoids as the strengthening phase [8,9,10]. Al–Mg–Si alloys intergranular corrosion (IGC) is mainly affected by the grain boundary composition and micro-galvanic coupling between Mg_2_Si precipitates as well as Mg, Si segregation at the grain boundary, and precipitate free zone (PFZ) along grain boundaries [7,11,12,13,14,15]. Xu et al. [16] showed that the IGC resistance of the alloy treated by ageing T616 improved, due to the alloy consists of discontinuously distributed grain boundary precipitates (GBPs), and narrow precipitation-free zones (PFZs) after being treated by ageing T616 compared to ageing T6 [17]. Research also found that the type, size, volume fraction, and distribution of β-phase (Mg_2_Si) changes can affect the alloy corrosion behaviour after ageing [7,13]. Mg_2_Si is anodic with respect to the substrate, and Mg preferential dissolution and simultaneous Si enrichment during corrosion. Mg_2_Si transforms from the anode to the cathode, leading to corrosion of the alloy base around the Mg_2_Si phases [11,15].

Recent research found that Mg_2_Si precipitates act as the anode phase to protect the Al matrix, and the severe segregation of AlFeSi phases accelerate the corrosion of matrix. The decrease of AlFeSi phases reduces the probability of micro-galvanic corrosion [18]. Corrosion behaviour variation was related to the formation of the micro-galvanic coupling between the Al matrix and the cathodic matrix grains with Fe-rich inclusions [5,19].

Intermetallics (precipitates, inclusions, and dispersoids) in Al are known to be primarily responsible for corrosion initiation [19], but enhance pitting development is not clear. In order to further understand the relationship between the second phase and Al–Mg–Si alloy corrosion behaviour. The second phase (AlFeSi/Mg_2_Si) which is dominant in the localised corrosion of 6061 aluminum alloy and their role in the corrosion behaviour of Al–Mg–Si alloys. In the present study, confocal laser scanning microscopy (CLSM), scanning Kelvin probe force microscopy (SKPFM), and electron probe microanalysis (EPMA) were employed to investigate the influence of the second phase on 6061 alloy corrosion behaviour at different ages.

## 2. Experiment

### 2.1. Materials

In the present study, 6061 cast aluminium alloy with a chemical composition of Mg 1.01 wt.%, Si 0.56 wt.%, Fe 0.21 wt.%, Cu 0.21 wt.%, Mn 0.03 wt.%, Zn 0.01 wt.%, Cr 0.18 wt.%, Ti 0.01 wt.%, and Al rem. was investigated. The Mg/Si ratios were approximately 1.804, which corresponded to a Mg-rich alloy.

### 2.2. Heat-Treatment Procedures and Hardness Testing

Temperature homogenization treatment at 570 °C for 7 h prompted the coarse second phase particles to dissolve into the matrix. After solution heat treatment at 550 °C for 1 h, the alloy was quenched immediately in a water bath at room temperature. These specimens were artificially aged at 180 °C at 0 h, 1 h, 2 h, 3 h, 4 h,5 h, and 14 h to obtain different Mg_2_ Si quantities and shapes. The 10 Vickers hardness numbers were measured on specimens aged at different times and the average hardness values were calculated.

### 2.3. Corrosion Testing

Before the corrosion test, the samples were ground with alcohol on SiC paper from 240 grade to 7000 grade, followed by mechanical polishing with polishing lapped cloth using a 0.5 μm diamond suspension. Subsequently, the specimens were cleaned ultrasonically in an acetone bath and anhydrous ethanol in turn and dried in a cool air stream. All corrosion tests were carried out in a 3.5% NaCl solution at a constant temperature 30 ± 1 °C.

#### 2.3.1. Electrochemical Corrosion Testing

Potentiodynamic polarization curve measurements were taken on an electrochemical work station (Model CS350, Corrtest, Wuhan, China). A self-designed three-electrode device was used for the electrochemical corrosion test. The polished specimens were used as working electrodes. The exposed area of the working electrode was 0.5 cm^2^. A saturated calomel was used as a reference electrode (SCE) and a Pt plate electrode was used as a counter electrode, respectively.

After the sample was immersed in the test solution for about 1 h until the sample surface reached a steady state, the potentiodynamic polarization curve was measured. The scan rate of potentiodynamic polarization curves is 0.5 mV·s^−1^. The potential scanning in the 3.5% NaCl electrolyte ranges from −0.08 V to 0.7 V versus open circuit potential (OCP). CVIEW 2 software was used to analyze the electrochemical experimental data.

#### 2.3.2. In Situ Observation by Confocal Laser Scanning Microscopy

Immersion corrosion testing was conducted at different time (0 h, 1 h, 8 h, 24 h, 72 h, 120 h and 168 h) in 3.5% NaCl solution at 30 ± 1 °C. Corrosion susceptibility was evaluated by examining the corroded samples surface. The second phase influence on corrosion behaviour by in situ observation performed by LEXT 3D MEASURING LASER MICROSCOPE OLS 5000.

### 2.4. Transmission Electron Microscopy (TEM) Measurements

Transmission electron microscopy (TEM) was used to observe the precipitation morphologies in the 6061 aluminium alloy matrix with different aging time. TEM analysis was conducted on transmission electron microscope (FEI Talos F200X). A Schottky thermal field-emission electron gun was selected for testing at an acceleration voltage of 200 kV. Before testing, the sample thinned by ion thinning after mechanical thinning to 50 μm. Digital micrograph software was used to analyze the TEM measurements data.

### 2.5. Scanning Kelvin Probe Force Microscopy (SKPFM) Measurements

#### 2.5.1. Sample Preparation

The smooth and clear surface of the sample used in the SKPFM measurement needed to be polished by argon ions after being mechanically polished. Prior to the SKPFM test, the samples needed ultrasonic cleaning in an anhydrous ethanol bath and dried in a constant temperature vacuum drying oven.

#### 2.5.2. Testing Parameters

SKPFM testing was conducted on a Bruker Dimension Icon scanning probe microscope to test the surface potential of second phase and matrix of 6061 aluminium alloy. The working principle and relevant knowledge of SKPFM have been reported in previous literature [20,21]. SKPFM measurements were performed with a pixel resolution of 256 × 256, and a scan frequency rate of 0.996 Hz in dry air at room temperature in this study. The tip reliability was evaluated by the volt potential of standard samples of gold and aluminium. In this study, the dual-scan mode was used. The first scan was used to obtain the surface topography of the sample, and the second scan with the probe lifted to 80 nm was used to obtain the surface Volta potential data. The bias voltage of 750 mV was held at the tip, and the volt potential diagrams were not inverted. NanoScope Analysis 1.5 software was used to analyze the SKPFM data. The height of the surface topography is the relative height, and the minimum value of the height value is set to zero by NanoScope Analysis 1.5 software.

### 2.6. Electron Probe Microanalysis (EPMA)

EPMA was used to analyze the element changes of Mg_2_Si phases and AlFeSi phases during the corrosion process. EPMA testing was performed on the equipment of JXA-8530F PLUS. Sample preparation is the same as Section 2.3. After mechanical polishing, the specimens were cleaned ultrasonically in an acetone bath and anhydrous ethanol bath and dried in a cool air stream. Then the samples were immersed in 3.5% NaCl solution at 30 ± 1 °C for 0 h, 1 h and 24 h, respectively. Before the test, samples were cleaned ultrasonically once again with anhydrous ethanol.

## 3. Results

### 3.1. Electrochemical Behaviour in 3.5% NaCl Solution

**Figure 1:**
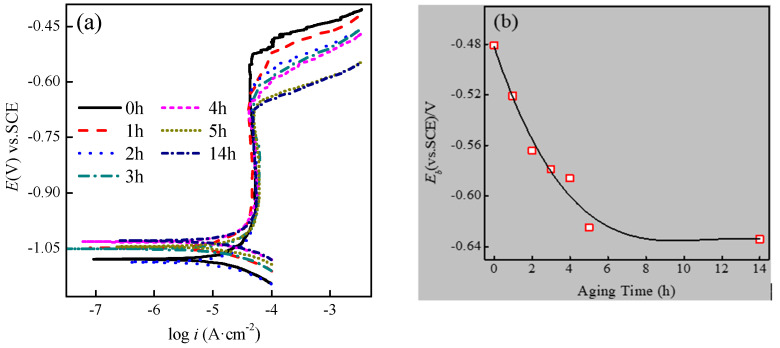
(**a**) Potentiodynamic polarization curves in 3.5% NaCl solution for samples aged for different times (1 h, 2 h, 3 h, 4 h, 5 h, and 14 h) at 180 °C; (**b**) breakdown potential change curve with aging time.

Potentiodynamic polarization curves in 3.5% NaCl solution at 30 ± 1 °C for samples aged at 180 °C for different times (1 h, 2 h, 3 h, 4 h, 5 h, 14 h) are shown in Figure 1a. The values of corrosion potential (*E*_corr_), the breakdown potential of passive film (*E_b_*) and the corrosion current density (*i*_corr_) can be obtained from the potentiodynamic polarization curves, as shown in Table 1. It is observed from Table 1 that the effect of aging time on E_corr_ is minor. However, the corrosion current density (*i*_corr_) increases with aging time, due to the existence and subsequent greater prevalence of Mg_2_Si phases [7,22].It can be clearly seen, the breakdown potential of passive film (*E_b_*) shifted in the negative with the ageing time before 5 h. The statistic results are shown in Figure 1b. As is well known, the 6061 aluminium alloy is a precipitation-hardening alloy. A high density of precipitates formed in peak-aged conditions (180 °C for 5 h) in the substrate. Therefore, it was speculated that the *E_b_* shifted in the negative direction was related to the formation of precipitates. However, as mentioned above, there were at least three types of second phase, which actually means *E_b_* negatively shift cannot be determined. It is important to note that the *E_b_* at 14 h was close to that at 5 h. Precipitates had nearly no effect on the *E_b_* negative shift of aluminium alloy at over the aged state (14 h) and the second phases reached peak in peak-aged condition (5 h). The breakdown potential of aluminum alloy clearly decreased within 5 h of the aged time, but the relationship between aged time and the passive current densities was not observed. According to the PDM (point defect model), the nature of the passivation film formed on the aluminium alloy surface did not change significantly with aging time. The aluminium alloy passivation film was mainly composed of Al_2_O_3_. The addition and precipitation of other substances primarily improved macro- defects density, but did not contribute to the aluminium alloy oxidation film composition [23,24].

### 3.2. Mg_2_Si Phase Characterization under Different Aged Times

#### 3.2.1. Age-Hardening Behaviour

**Figure 2:**
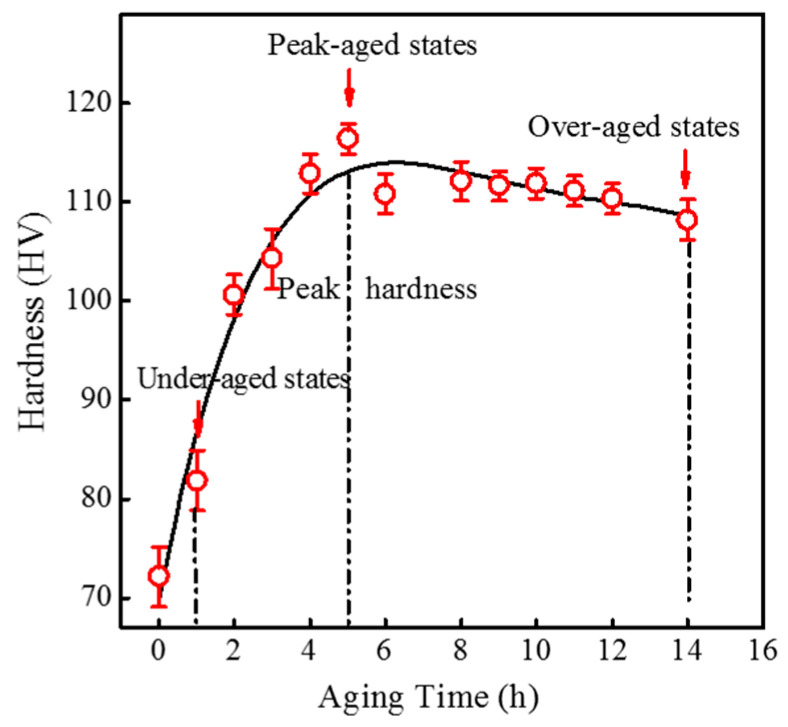
Variation in hardness as a function of ageing time for samples aged at 180 °C after solution heat treatment at 550 °C for 1 h.

Figure 2 shows the hardness curves of the 6061 aluminium alloy aged at 180 °C at different times immediately after water quenching. Compared with the samples after solid solution treatment, the 180 °C aging treatment significantly enhanced hardness, and it increased gradually with aged time with a peak value of 116.4 ± 1.5 HV at 5 h. The hardness then decreased slowly with increasing aged time, while the main strengthening stage in the peak aged stage is β”. According to the previous literature [25,26,27], the rapid hardness increase was mainly due to clusters and GP zones formation at the under-aged stage, while the main strengthening phase at peak aged stage was β″. Thus, under aged (1 h), peak aged (5 h), and overaged (14 h) conditions were selected to study the precipitate evolution.

#### 3.2.2. TEM Investigation

**Figure 3:**
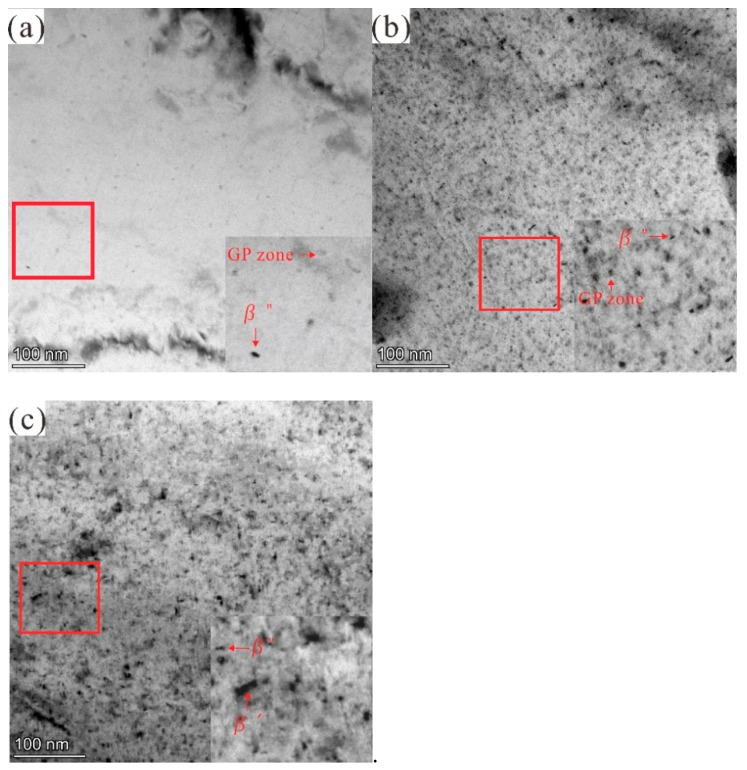
Transmission electron microscopy (TEM) bright-field images of the 6061 aluminium alloy aged at 180 °C for different times: (**a**) 1 h, (**b**) 5 h, (**c**) 14 h.

**Figure 4:**
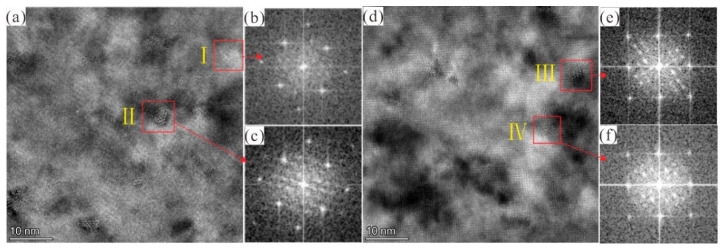
Representative high-resolution image of the primary strengthening phase in the Al–Mg–Si alloy. (**a**) Overviews of the precipitated particles in the samples under the 180 °C peak-aged condition. (**b**,**c**) corresponding FFT patterns of the GP zone and β” precipitates corresponding to zone I and zone II in (**a**), respectively. (**d**) Overviews of the precipitated particles in the samples under the 180 °C overaged condition. (**e**,**f**) Corresponding FFT patterns of the β” and β’ precipitates corresponding to zone III and zone IV in (**d**), respectively.

To verify the internal relationship between 6061 aluminium alloy hardness and microstructure under experimental conditions, according to the hardness curve, specimens at the under-aged states (1 h), peak aged states (5 h) and overaged states (14 h) were selected for a bright field image of TEM, as shown in Figure 3a–c. High-resolution TEM (HRTEM) images of precipitate at peak aged states and overaged states and the corresponding Fast Fourier transforms (FFT)pattern are shown in Figure 4.

It is generally believed that the precipitation sequence of Al–Mg–Si alloys is the following: supersaturated solid solution → clusters/GP zones → β” *→* β’ (B’/U1/U2) → β [3,8,9]. Figure 3a shows that the alloy had a large number of fine and uniformly distributed point-like precipitation phases (GP zone) in the under-aged (1 h) state. Due to the ordered structure within the precipitates, these precipitates were identified as GP regions (Figure 4b), which were precursors of the β” phase [26]. The precipitates formed in peak aged state (5 h) are shown in Figure 3b and Figure 4a–c, and the high density of fine needle-like precipitated phases (β”) can be clearly observed. During the aging treatment process, the needle-like precipitation *β*” phase in the aluminium alloy matrix could effectively prevent the dislocation movement, increasing the Al–Mg–Si alloy hardness [28]. The overaged (14 h) conditions alloy microstructure is shown in Figure 3c. Uniformly distributed rod-like precipitates were formed, which were identified as β″, and β’ phases from the HRTEM image and its corresponding FFT patterns presented in Figure 4d–f.

The TEM results showed that the amount of nanosize Mg_2_Si precipitated from the aluminium alloy substrate was relatively obvious during the aging process from the results. Some researchers [7] thought that corrosion behaviour was closely related to nanosize precipitates. However, it is not known whether the increase in the number of nanosize precipitates is necessarily related to aluminium alloy corrosion resistance and requires further study.

### 3.3. AlFeSi Phase Characterization

**Figure 5:**
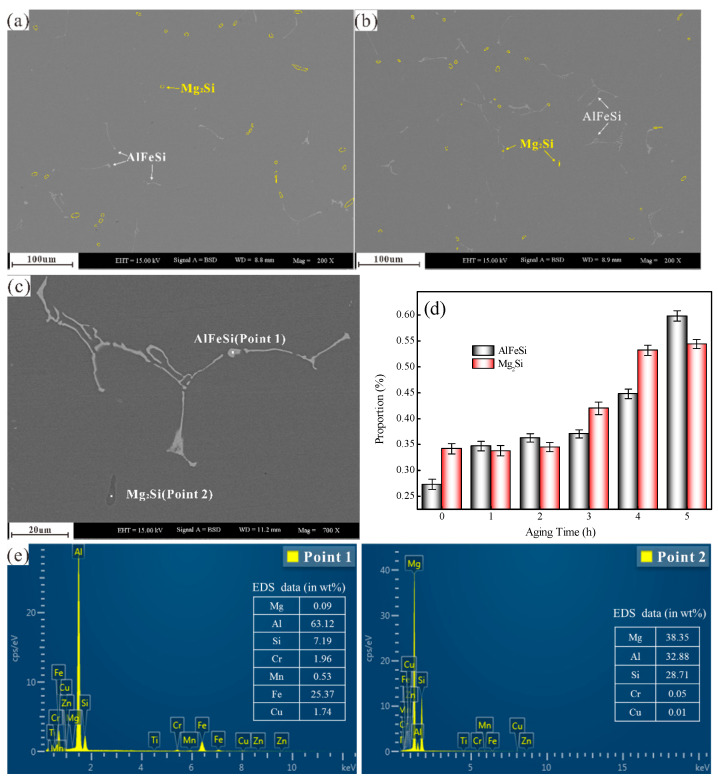
Scanning electron microscopy (SEM) analyses of second phases in different artificial ageing times at 180 °C: (**a**)1 h, (**b**) 5 h, (**c**) local enlarged view of second phases aged at 180 °C for 5 h, (**d**) volume fraction of the AlFeSi phase and Mg_2_Si phase under different ageing time (0 h, 1 h, 2 h, 3 h, 4 h and 5 h), and (**e**) energy-dispersive X-ray spectroscopy (EDS) analyses of (**c**).

Figure 5a,b shows the BSD pattern of SEM analyses of second phases at 180 °C aged at 1 h and 5 h, respectively. The microsize second phase is mainly the light AlFeSi phases and dark Mg_2_Si phases in Figure 5c,e. The AlFeSi phase existed in the shape of fish bone, block, and needle shapes, and the Mg_2_Si phase marked by the yellow line existed as disk and rod shapes along the grain boundaries. Figure 5d shows AlFeSi phase and microsize Mg_2_Si phase volume fraction changes at different artificial aged times (1–5 h) at 180 °C through image Pro Plus statistics and analysis. It was found that the proportion of AlFeSi phase and microsize Mg_2_Si phase in the matrix increased gradually from 1 h to 5 h. Nevertheless, Alvarez-Antolin et al. [29] pointed out that AlFeSi intermetallics can undergo phase transformation and change their morphology during homogenization, but they are insoluble during solution heat treatment and aged stage in Al–Mg–Si alloys. The reason for the AlFeSi phase increase with aged time needs to be further studied. The statistical results (Figure 5d) also showed that the AlFeSi and microsizeMg_2_Si phase proportion were nearly similar. As to which of second phases is dominant in the localised corrosion of 6061 aluminium alloy, further research is needed.

### 3.4. SKPFM-Measured Volta Potential Map

**Figure 6:**
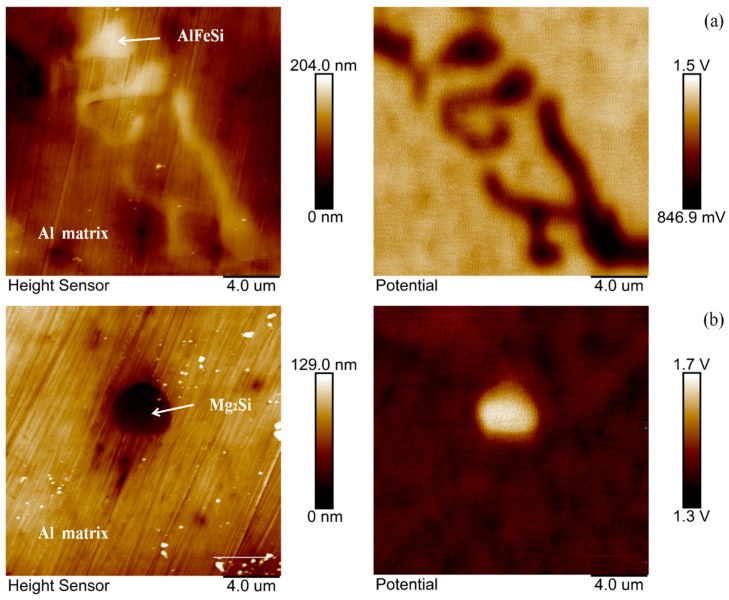

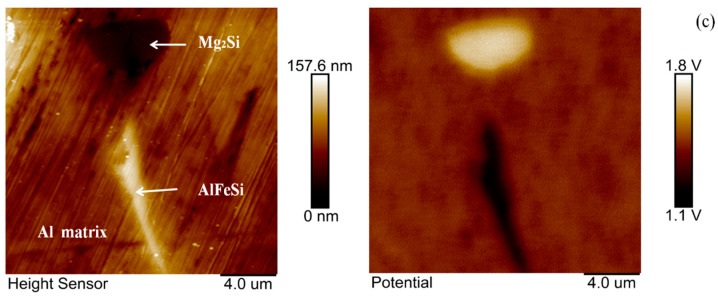
Topography and Volta potential maps of different second phases aged at 180 °C for 5 h: (**a**) AlFeSi phase and matrix, (**b**) Mg_2_Si phase and matrix, and (**c**) AlFeSi phase, Mg_2_Si phase and matrix.

The Volta potential is a material specific property and used to evaluate the (relative) noble metal properties of the local microstructure of an alloy. It describes the driving force of a metal used in chemical or electrochemical reactions. If the tip is biased, low potential indicates that the cathodic region is protected, while high potential indicates that the anodic area is susceptible to corrosion [30]. Therefore, a high Volta potential indicates a low electrochemical nobility, and thus, the Volta potential value can be considered a standard for predicting corrosion behaviour. The Volta potential of Mg_2_Si and AlFeSi was characterized by SKPFM. Topography and Volta potential maps of different second phase aged after 5 h, as shown in Figure 6.

As the AFM image was based on the height information, a representative fish- bone like morphology was chosen to ensure that the AlFeSi phase was measured (Figure 6a). As can be seen, the Volta potential of AlFeSi phase was 846.9 mV lower than that of Al substrate, suggesting that the AlFeSi phase was much more noble. Hence, from a galvanic corrosion point of view, the nobler phase played the role of cathodic region according to the microcell corrosion theory. A representative microsize Mg_2_Si phase was also selected as shown in Figure 6b. It can be seen, the Volta potential of Mg_2_Si was 1.7 V higher than that of Al substrate, indicating an anodic (less noble) nature relative to the matrix. Interestingly, a region was found where the Mg_2_Si phase and AlFeSi phase existed simultaneously. According to Figure 6c, the AlFeSi phase exhibited the lowest Volta potential, while the highest Volta potential corresponded to the Mg_2_Si phase. The Al matrix Volta potential was between that of the AlFeSi phase and Mg_2_Si phase. Therefore, the AlFeSi phases were cathodic with respect to the matrix and promoted matrix dissolution, while the Mg_2_Si phases were anodic relative to the matrix and dissolved preferentially.

### 3.5. In Situ Observation by Confocal Laser Scanning Microscopy

**Figure 7:**
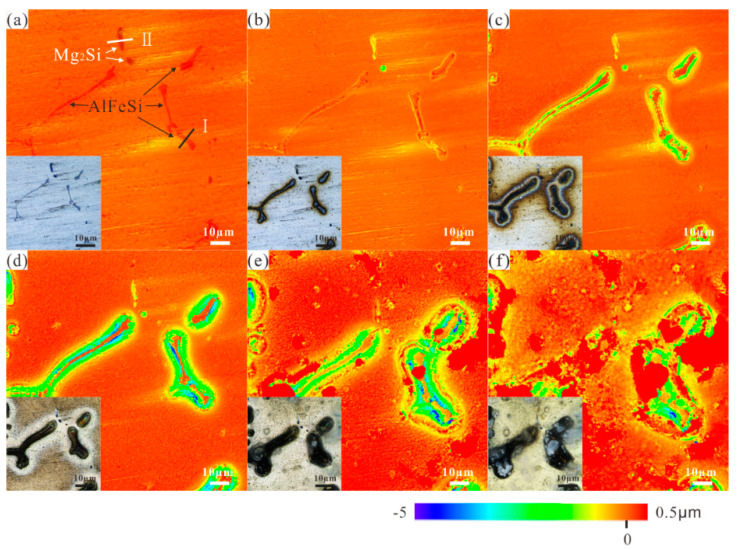
Corrosion morphologies of Al alloys aged 5 h at 180 °C immersed in 3.5% NaCl solution for different times: (**a**) 0 h, (**b**) 1 h, (**c**) 8 h, (**d**) 24 h, (**e**) 72 h, and (**f**) 120 h.

**Figure 8:**
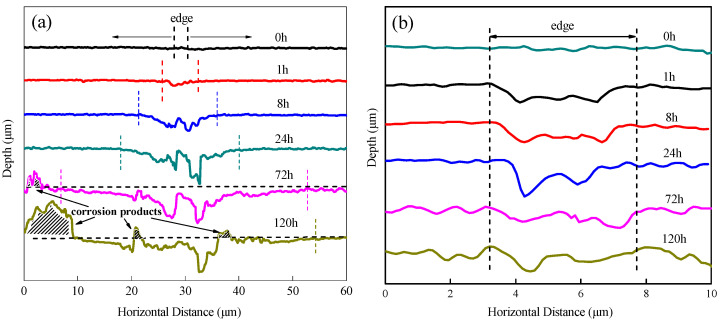
Cross section width and depth change where the AlFeSi phase is located (Figure 7a-I) and the position where the Mg_2_Si phase is located (Figure 7a-II): (**a**) AlFeSi phase, and (**b**) Mg_2_Si phase.

**Figure 9:**
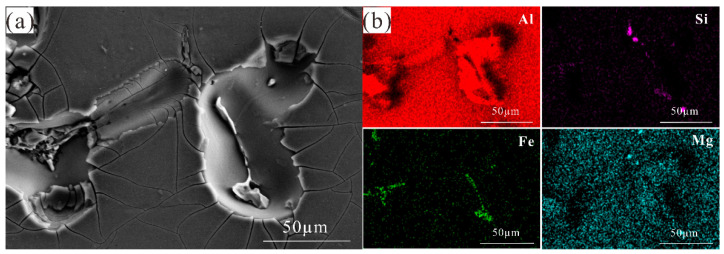
(**a**) SEM analyses of corrosion morphology after the corrosion products were removed obtained by immersion in 3.5% NaCl solution after 168 h; (**b**) EDX elemental maps of (**a**).

In situ observation by laser confocal microscopy was used to study the influence of the AlFeSi, microsizeMg_2_Si and nanosize Mg_2_Si phases on the 6061 aluminium alloy localised corrosion. Figure 7 displays corrosion morphologies of 6061 aluminium alloy aged 5 h at 180 °C immersed in 3.5% NaCl solution for different times (0 h, 1 h, 8 h, 24 h, 72 h, and 120 h). Figure 8 shows the change in cross section width and height where Mg_2_Si (Figure 7a-I) and AlFeSi (Figure 7a-II) at different immersed times. After immersion for 168 h, the corrosion products were removed, and the corrosion morphology is shown in Figure 9.

It clearly observed that the Al substrate dissolved preferentially near the AlFeSi phase, and the corrosion range expanded with the immersion time, as shown in Figure 7; Figure 8a. Compared with the matrix, the AlFeSi phase was a cathode phase (Figure 6), which promoted the corrosion of the surrounding aluminium matrix. The Al matrix had a cathodic protection effect on the AlFeSi phase. However, with the dissolution of Al matrix around the AlFeSi phase and pit expansion, the resistance between the AlFeSi phase and the solution increased. After reaching the limit, the AlFeSi phase lost the Al matrix protection, and AlFeSi phase started to dissolve. The AlFeSi phase dissolved with immersion time, as shown in Figure 9. Therefore, there was limited influence from the AlFeSi phase, and the AlFeSi phase influence width was at least five times that of itself.

However, it was found that the Mg_2_Si phases dissolved preferentially after immersion in 3.5% NaCl solution after 1 h, as shown in Figure 7; Figure 8b). Mg_2_Si phases were anodic with respect to the matrix (Figure 6) and dissolved preferentially. The Mg_2_Si phases formed pits by initial rapid Mg dissolution and Si enrichment (Figure 9). It is worth noting that the pit depth did not change after 1 h and before 8 h, but deepened at 24 h. This was because Si particles led to Al substrate dissolution, as the corrosion time was extended [11]. After 24 h, the corrosion products began to accumulate.

Hence, the AlFeSi phase significantly affected 6061 aluminium alloys corrosion behaviour.

### 3.6. EPMA Element Distribution Characterisation

**Figure 10:**
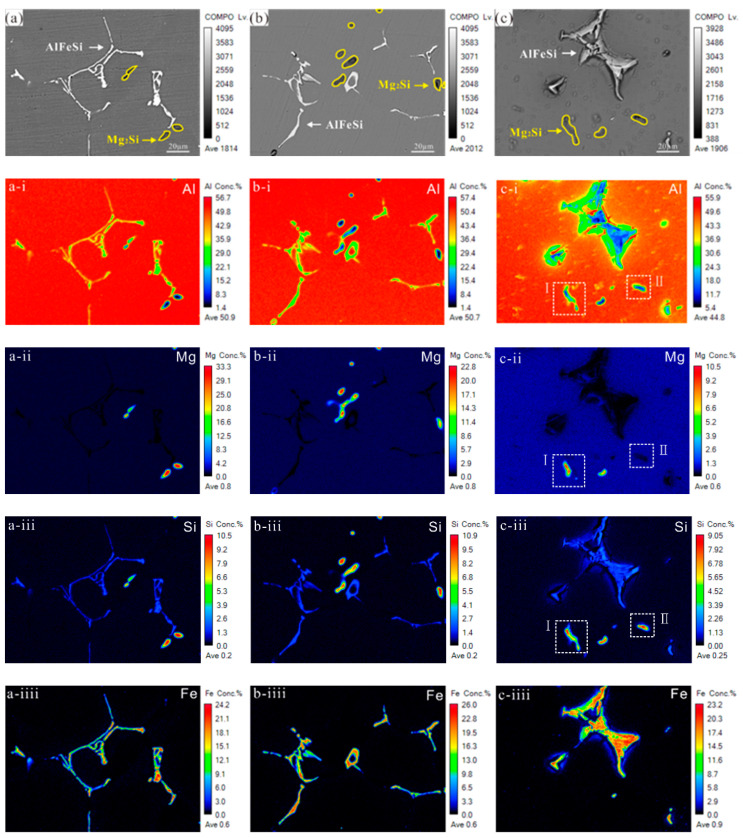

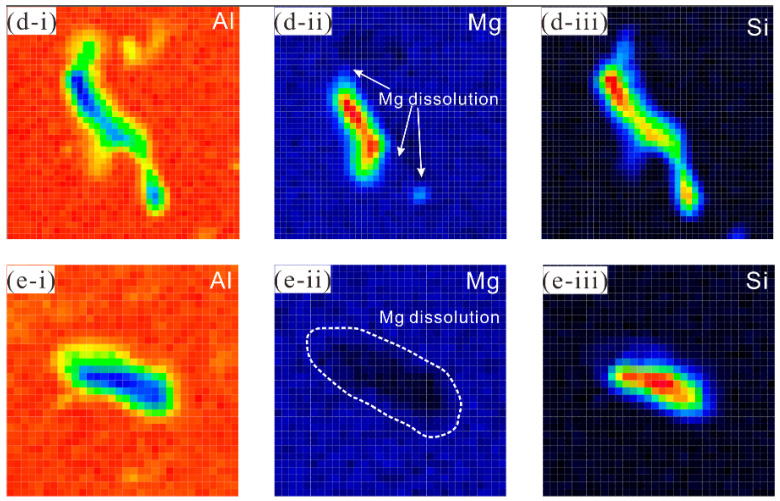
The change in element content (Al, Fe, Si, Mg) of samples aged 5 h at 180 °C immersed in 3.5% NaCl solution for different times: (**a**)–(**a-iiii**) 0 h; (**b**)–(**b-iiii**) 1 h; and (**c**)–(**c-iiii**) 24 h. (**d-i**)–(**d-iii**) and (**e-i**)–(**e-iii**) are enlarged images of regions (**I**) and (**II**) in (**c-i**) and (**c-ii**), respectively.

The Al, Fe, Si and Mg element contents after immersion in 3.5% NaCl solution for 0 h, 1 h and 24 h were investigated by EPMA (Figure 10) to further analyze the Mg_2_Si phase and AlFeSi phase changes during corrosion.

Figure 10 shows that Fe content was more than 20%, while Si only accounted for approximately 10%. At the same time, it was verified that the AlFeSi phases were in fish bone, block, and needle shapes. Hence, the 6061 aluminium alloy local corrosion was mainly affected by Fe. Mg partly dissolved when immersed for 1 h. After immersion for 24 h, Mg also partly dissolved in some regions, as shown in Figure 10d-ii. However, in some regions, Mg dissolved completely, as shown in Figure 10e-ii. One possible explanation for this is that Mg in some Mg_2_Si phases was exposed on the surface, so Mg were completely dissolved. Mg was covered by Si in some Mg_2_Si phases, resulting in partial Mg dissolution.

## 4. Discussion

**Figure 11:**
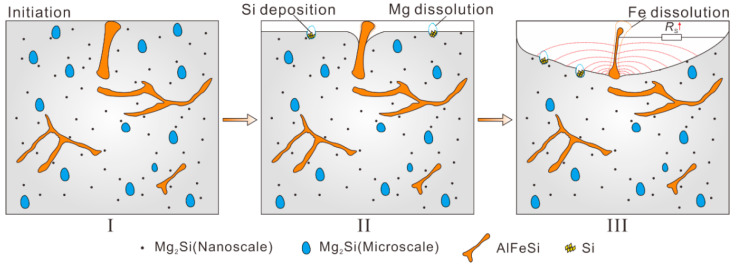
Schematic diagram of the functional mechanism of the AlFeSi phase, microsize Mg_2_Si phase and nanosizeMg_2_Si phase in 6061 aluminium alloy localized corrosion.

The 6061 aluminium alloy could be seen as a composite material made of pure aluminium, nanosize Mg_2_Si, microsize Mg_2_Si and AlFeSi, as shown in Figure 11-I. Although nanosize Mg_2_Si phases widely existed in the substrate, from an electrochemical point of view, nanosize Mg_2_Si hardly formed large local corrosion microcells as microsize Mg_2_Si phases do. Instead, nanosizeMg_2_Si mainly affected the Al substrate exchange current density as nanosize Mg_2_Si uniformly precipitated in the substrate. Therefore, the main factor influencing the localised corrosion was the second phase on the microsize, i.e., the remaining microsize Mg_2_Si and AlFeSi phases [6].

Interestingly, the Mg_2_Si and AlFeSi polarities were opposite. The phases that were more noble than the substrate acted as cathodes to accelerate substrate dissolution, while less noble phases such as Mg_2_Si acted as anodes that were preferentially dissolved [11,31]. In terms of the contribution to corrosion current, the increased anodic phase ratio negatively shifted the alloy potential and enlarged the corrosion current; however, the anodic dissolution process mainly occurred on the microsize Mg_2_Si phases. As far as the experimental results presents (as shown in Figure 7 and Figure 10), microsize Mg_2_Si phase duration was relatively short; thus, the preferentially dissolved microsize Mg_2_Si could not have a persistent effect on the alloy. However, Zeng et. al. [11] found that Si in Mg_2_Si was enriched after Mg dissolved. Hence, the remaining Si particles accelerated adjacent Al substrates corrosion, as shown in Figure 11-II. Nevertheless, compared with the galvanic effect induced by larger AlFeSi phases, the Si particle contributed a minor effect.

During the corrosion process, the AlFeSi phase always acts as a cathodic zone. It was observed that the Al substrate dissolved preferentially near the AlFeSi phase, and the corrosion range expanded with immersion time (Figure 11-II). The AlFeSi phase had a flake structure, which was partly on the metal surface and partly inside the metal [32,33]. As shown in Figure 11-III, with extended immersion time, the influence range of AlFeSi phase reached its maximum, but AlFeSi also dissolved (Figure 9). This phenomenon suggested that the aluminium substrate cathodic protection effect weakened with pit expansion. In other words, the distance between the AlFeSi flake and the aluminium substrate increased the solution resistance. Therefore, when the corroded region width reached five times of the AlFeSi flake, the accelerating effect could be terminated as the AlFeSi dissolved. Hence, it is believed that aluminium alloy local corrosion was mainly affected by AlFeSi phases.

## 5. Conclusions

In situ corrosion experiments in 3.5% NaCl solution on 6061 aluminium alloy were performed using a confocal laser scanning microscope, and the individual effects of different phases on localised corrosion were investigated.

(1) The breakdown potential of passive film (*E_b_*) shifted in the negative with the aging time was found by electrochemical experiments.

(2) The microsize Mg_2_Si precipitates dissolved quickly during corrosion leading to enrichment of the 6061 alloy matrix in Si.

(3) The AlFeSi precipitates exerted the longer-range influence on the localised corrosion of the 6061 aluminium alloy.

(4) The extent of the localised corrosion of the aged 6061 aluminium alloy was mainly affected by the AlFeSi phase.

## Figures and Tables

**Table 1:** Electrochemical parameters of the samples in 3.5% NaCl solution, extracted from potentiodynamic polarization curves fitted in Tafel regions.

Aging Time (h)	*E*_corr_ (V)	*i*_corr_ (μA/cm^2^)	*β_a_* (mV)	*β_c_* (mV)	Corrosion Rate (mm/a)
0	−1.079	24.395	174.241	78.745	0.744
1	−1.049	24.340	326.920	84.048	0.743
2	−1.087	28.385	235.262	92.201	0.866
3	−1.051	29.491	172.450	90.886	0.900
4	−1.032	31.576	244.220	76.776	0.963
5	−1.045	34.239	198.951	83.748	1.045
14	−1.029	34.723	232.833	84.178	1.059

## Data Availability

The data presented in this study are available on request from the corresponding author.

## References

[1] Kolobnev L.B.B.N.I., Khokhlatova L.B., Ryabov D.K. (2012). Structure, Properties and Application of Alloys of the Al–Mg–Si–(Cu) System. Met. Sci. Heat Treat..

[2] Cui L., Guo M., Peng X., Zhang Y., Zhang J., Zhuang L. (2015). Influence of Pre-deformation on the Precipitation Behaviors of Al-Mg-Si-Cu Alloy for Automotive Application. Acta Metall. Sin..

[3] Lai Y., Fan W., Yin M., Wu C., Chen J. (2020). Structures and formation mechanisms of dislocation-induced precipitates in relation to the age-hardening responses of Al-Mg-Si alloys. J. Mater. Sci. Technol..

[4] Zhong H., Rometsch P., Estrin Y. (2014). Effect of alloy composition and heat treatment on mechanical performance of 6xxx aluminum alloys. Trans. Nonferrous Met. Soc. China.

[5] Zander B.D., Schnatterer C., Altenbach C., Chaineux V. (2015). Microstructural impact on intergranular corrosion and the mechanical properties of industrial drawn 6056 aluminum wires. Mater. Des..

[6] Blanc C., Mankowski G. (1997). Susceptibility to pitting corrosion of 6056 aluminium alloy. Corros. Sci..

[7] El-Menshawy K., El-Sayed A.-W.A., El-Bedawy M.E., Ahmed H.A., El-Raghy S.M. (2012). Effect of aging time at low aging temperatures on the corrosion of aluminum alloy 6061. Corros. Sci..

[8] van Huis M., Chen J., Zandbergen H., Sluiter M. (2006). Phase stability and structural relations of nanometer-sized, matrix-embedded precipitate phases in Al–Mg–Si alloys in the late stages of evolution. Acta Mater..

[9] Chen H., Lu J., Kong Y., Li K., Yang T., Meingast A., Yang M., Lu Q., Du Y. (2020). Atomic scale investigation of the crystal structure and interfaces of the B′ precipitate in Al-Mg-Si alloys. Acta Mater..

[10] Edwards G.A., Stiller K., Dunlop G.L., Couper M.J. (1998). The precipitation sequence in Al–Mg–Si alloys. Acta Mater..

[11] Zeng F.-L., Wei Z.-L., Li J.-F., Li C.-X., Tan X., Zhang Z., Zheng Z.-Q. (2011). Corrosion mechanism associated with Mg2Si and Si particles in Al–Mg–Si alloys. Trans. Nonferrous Met. Soc. China.

[12] Kairy S., Rometsch P., Davies C., Birbilis N. (2017). On the Intergranular Corrosion and Hardness Evolution of 6xxx Series Al Alloys as a Function of Si:Mg Ratio, Cu Content, and Aging Condition. Corrosion.

[13] Zeid E.F.A. (2019). Mechanical and electrochemical characteristics of solutionized AA 6061, AA6013 and AA 5086 aluminum alloys. J. Mater. Res. Technol..

[14] Zhang X., Zhou X., Nilsson J.-O. (2019). Corrosion behaviour of AA6082 Al-Mg-Si alloy extrusion: The influence of quench cooling rate. Corros. Sci..

[15] Yoshida T.M. (2002). Effect of grain boundary characteristics on intergranular corrosion resistance of 6061 aluminum alloy extrusion. Metall. Mater. Trans. A.

[16] Xuehong X., Deng Y., Shuiqing C., Xiaobin G. (2020). Effect of interrupted ageing treatment on the mechanical properties and intergranular corrosion behavior of Al-Mg-Si alloys. J. Mater. Res. Technol..

[17] Wang Z., Li H., Miao F., Sun W., Fang B., Song R., Zheng Z. (2014). Improving the intergranular corrosion resistance of Al–Mg–Si–Cu alloys without strength loss by a two-step aging treatment. Mater. Sci. Eng. A.

[18] Zhu M., Zhao B.Z., Yuan Y.F., Guo S.Y., Pan J. (2020). Effect of Solution Temperature on the Corrosion Behavior of 6061-T6 Aluminum Alloy in NaCl Solution. J. Mater. Eng. Perform..

[19] Ahmad Z. (2012). Aluminium Alloys—New Trends in Fabrication and Applications. Alum. Alloys New Trends Fabr. Appl..

[20] Cheng T., Shi W., Xiang S., Ballingerc R.G. (2020). Volta potential mapping of the gradient strengthened layer in 20CrMnTi by using SKPFM. J. Mater. Sci..

[21] Cook A., Barrett Z., Lyon S., McMurray H., Walton J., Williams G. (2012). Calibration of the scanning Kelvin probe force microscope under controlled environmental conditions. Electrochim. Acta.

[22] Liang W., Rometsch P., Cao L., Birbilis N. (2013). General aspects related to the corrosion of 6xxx series aluminium alloys: Exploring the influence of Mg/Si ratio and Cu. Corros. Sci..

[23] Ezuber H., El-Houd A., El-Shawesh F. (2008). A study on the corrosion behavior of aluminum alloys in seawater. Mater. Des..

[24] Vallabhaneni R., Stannard T.J., Kaira C.S., Chawla N. (2018). 3D X-ray microtomography and mechanical characterization of corrosion-induced damage in 7075 aluminium (Al) alloys. Corros. Sci..

[25] Weng Y., Ding L., Zhang Z., Jia Z., Wen B., Liu Y., Muraishi S., Li Y., Liu Q. (2019). Effect of Ag addition on the precipitation evolution and interfacial segregation for Al–Mg–Si alloy. Acta Mater..

[26] Weng Y., Ding L., Jia Z., Liu Q. (2021). Effect of combined addition of Ag and Cu on the precipitation behavior for an Al-Mg-Si alloy. Mater. Charact..

[27] Lu Q., Li K., Chen H., Yang M., Lan X., Yang T., Liu S., Song M., Cao L., Du Y. (2020). Simultaneously enhanced strength and ductility of 6xxx Al alloys via manipulating meso-scale and nano-scale structures guided with phase equilibrium. J. Mater. Sci. Technol..

[28] Yang W., Huang L., Zhang R., Wang M., Li Z., Jia Y., Lei R., Sheng X. (2012). Electron microscopy studies of the age-hardening behaviors in 6005A alloy and microstructural characterizations of precipitates. J. Alloy. Compd..

[29] Alvarez-Antolin F., Asensio-Lozano J., Cofiño-Villar A., Gonzalez-Pociño A. (2020). Analysis of Different Solution Treatments in the Transformation of β-AlFeSi Particles into α-(FeMn)Si and Their Influence on Different Ageing Treatments in Al–Mg–Si Alloys. Metals.

[30] Örnek C., Leygraf C., Pan J. (2019). On the Volta potential measured by SKPFM—fundamental and practical aspects with relevance to corrosion science. Corros. Eng. Sci. Technol..

[31] Wang Y., Deng Y., Chen J., Dai Q., Guo X. (2020). Effects of grain structure related precipitation on corrosion behavior and corrosion fatigue property of Al–Mg–Si alloy. J. Mater. Res. Technol..

[32] Bacaicoa I., Luetje M., Wicke M., Geisert A., Zeismann F., Fehlbier M., Brueckner-Foit A. (2016). 3D Morphology of Al5FeSi inclusions in high Fe-content Al-Si-Cu Alloys. Procedia Struct. Integr..

[33] Brito C., Vida T., Freitas E., Cheung N., Spinelli J.E., Garcia A. (2016). Cellular/dendritic arrays and intermetallic phases affecting corrosion and mechanical resistances of an Al–Mg–Si alloy. J. Alloy. Compd..

